# Association Between Vascular Index Measured *via* Superb Microvascular Imaging and Molecular Subtype of Breast Cancer

**DOI:** 10.3389/fonc.2022.861151

**Published:** 2022-03-21

**Authors:** Xiao-Yan Zhang, Si-Man Cai, Li Zhang, Qing-Li Zhu, Qiang Sun, Yu-Xin Jiang, Hong-Yan Wang, Jian-Chu Li

**Affiliations:** ^1^ Department of Diagnostic Ultrasound, Peking Union Medical College Hospital, Chinese Academy of Medical Sciences, Beijing, China; ^2^ Department of Breast Surgery, Peking Union Medical College Hospital, Chinese Academy of Medical Sciences, Beijing, China

**Keywords:** breast cancer, molecular subtype, ultrasonography, superb microvascular imaging, vascular index

## Abstract

**Background:**

To determine whether vascular index (VI; defined as the ratio of Doppler signal pixels to pixels in the total lesion) measured *via* superb microvascular imaging in breast cancer correlates with immunohistochemically defined subtype and is able to predict molecular subtypes.

**Methods:**

This prospective study involved 225 patients with 225 mass-type invasive breast cancers (mean size 2.6 ± 1.4 cm, range 0.4~5.9 cm) who underwent ultrasound and superb microvascular imaging (SMI) at Peking Union Medical College Hospital before breast surgery from December 2016 to June 2018. The correlations between primary tumor VI measured *via* SMI, clinicopathological findings, and molecular subtype were analyzed. The performance of VI for prediction of molecular subtypes in invasive breast cancer was investigated.

**Results:**

The median VI of the 225 tumors was 7.3% (4.2%~11.8%) (range 0%~54.4%). Among the subtypes of the 225 tumors, 41 (18.2%) were luminal A, 91 (40.4%) were luminal B human epidermal growth factor receptor-2 (HER-2)-negative, 26 (11.6%) were luminal B HER-2-positive, 17 (7.6%) were HER-2-positive, and 50 (22.2%) were triple-negative, and the corresponding median VI values were 5.9% (2.6%~11.6%) (range 0%~47.1%), 7.3 (4.4%~10.5%) (range 0%~29.5%), 6.3% (3.9%~11.3%) (range 0.6%~22.2%), 8.2% (4.9%~15.6%) (range 0.9%~54.4%), and 9.2% (5.1%~15.3%) (range 0.7%~32.9%), respectively. Estrogen receptor (ER) negativity, higher tumor grade, and higher Ki-67 index (≥20%) were significantly associated with a higher VI value. Tumor size, ER status, and Ki-67 index were shown to independently influence VI. A cutoff value of 4.1% yielded 79.9% sensitivity and 41.5% specificity with an area under the receiver operating characteristic curve (AUC) of 0.58 for predicting that a tumor was of the luminal A subtype. A cutoff value of 16.4% yielded 30.0% sensitivity and 90.3% specificity with an AUC of 0.60 for predicting a triple-negative subtype.

**Conclusions:**

VI, as a quantitative index obtained by SMI examination, could reflect histologic vascular changes in invasive breast cancer and was found to be higher in more biologically aggressive breast tumors. VI shows a certain degree of correlation with the molecular subtype of invasive breast cancer and plays a limited role in predicting the luminal A with high sensitivity and triple-negative subtype with high specificity.

## Introduction

Breast cancer is the cancer type with the highest prevalence and the second highest cancer-related premature mortality rate among women worldwide (1). According to the 12th St. Gallen International Expert Consensus, breast cancer is categorized into five subtypes: luminal A, luminal B human epidermal growth factor receptor-2 (HER-2)-positive, luminal B HER-2-negative, HER-2-enriched, and triple-negative based on the expression status of the estrogen receptor (ER), progesterone receptor (PR), HER-2-positive, and Ki-67 index (2). Owing to the different molecular classifications, along with tumor size, tumor grade, and nodal status, breast cancer is a heterogeneous disease in biological behavior and prognosis (3). ER/PR-positive cancers are usually low grade and less aggressive. Luminal A and B cancers account for approximately 70% of breast cancers with positive hormone receptors. Generally speaking, luminal A cancers are low grade with the best prognosis among all subtypes. Luminal B cancers tend to be higher grade and have a worse prognosis than luminal A. HER-2 overexpression is associated with aggressive clinical course and poor prognosis (4). Triple-negative breast cancers (TNBCs), which accounted for about 15%–20% of breast cancers (5), are in general high grade and associated with a poor prognosis (6).

The formation of neovascularization and the increase of blood flow are the basis of cancer cell growth. Tumors cannot exceed 2 mm^3^ without vascular support (7). Angiogenesis in breast cancer is a well-established driver of cancer aggressiveness, therapy resistance, and poor prognosis (8, 9). Color Doppler ultrasound (US) imaging is the primary noninvasive modality for the vasculature evaluation of breast lesions. High-grade tumors usually have abundant vasculature, while low-grade tumors may have no demonstrable vascularization on Doppler US (10). Superb microvascular imaging (SMI; Canon Medical Systems) is a novel feasible microflow imaging technique applying multidimensional wall filtering systems that could improve sensitivity for low-flow tiny vessels and quantitatively assess tumor vascularity *via* measuring vascular index (VI) without the injection of contrast agents. VI was defined as the ratio of Doppler signal pixels to pixels in the total lesion measured *via* SMI. VI measured *via* SMI was a highly reproducible and objective quantitative parameter to estimate the degree of vascularity in breast lesions (11). Increased microvessel proliferation, an indicator of angiogenesis, was significantly correlated with negative ER status and basal-like phenotype in breast cancer (12–15). Previous studies showed that VI values of malignant breast lesions were significantly higher than those in benign breast lesions, and the combination of VI values with conventional B-mode US can enhance the diagnostic performance in differentiating benign from malignant breast masses (11, 16–19). However, the correlation between VI measured by SMI and the molecular subtypes in invasive breast cancer and the performance of VI for prediction of molecular subtypes in invasive breast cancer has not been investigated.

Therefore, the purpose of this study was to determine 1) whether a correlation exists between VI measured *via* SMI and the molecular subtype of invasive breast cancer defined by the St. Gallen International Expert Consensus and 2) whether VI could predict the molecular subtype of the invasive breast cancer.

## Materials and Methods

### Patients

**Figure 1:**
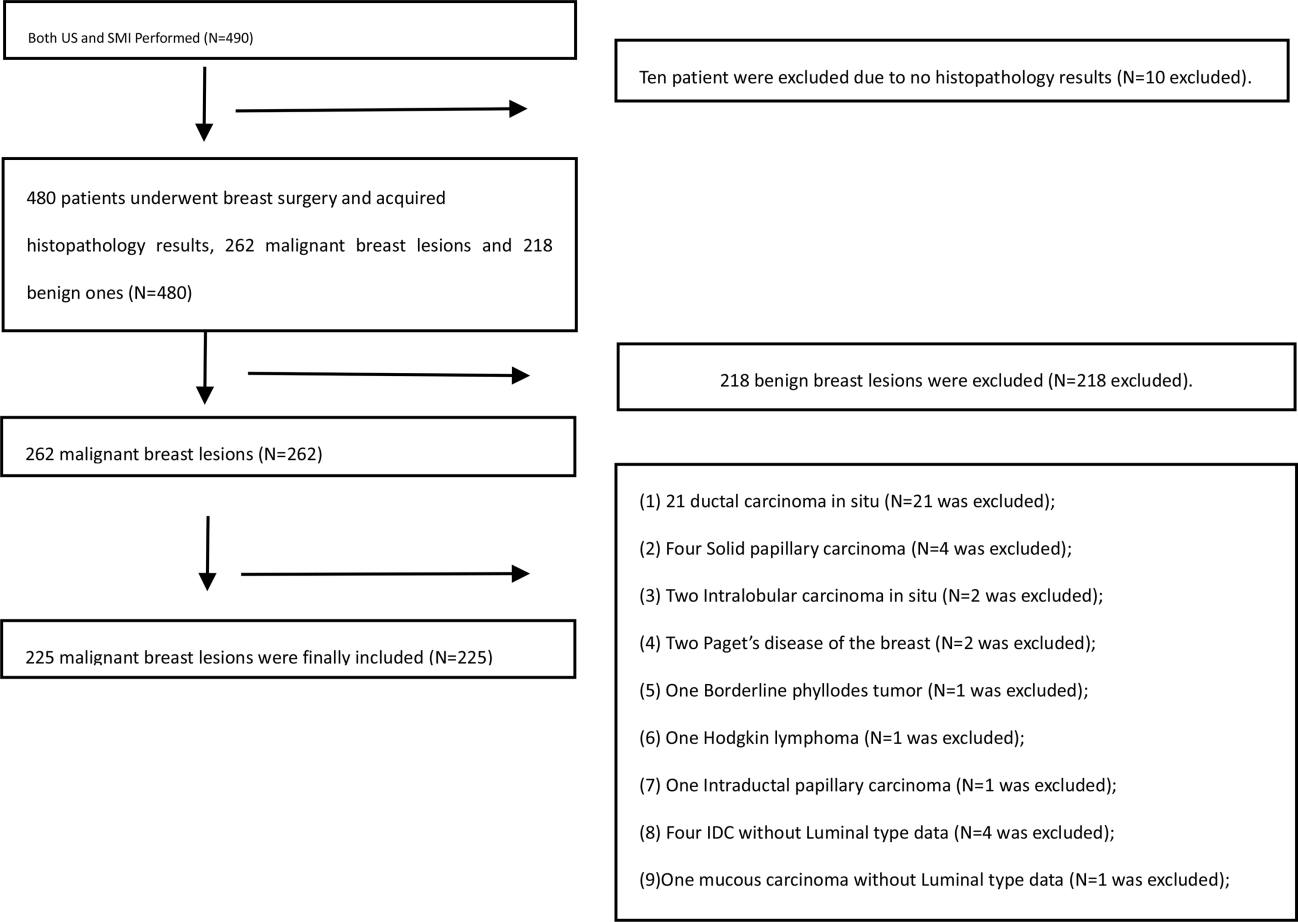
Flowchart of patient selection and inclusion in the study.

The institutional review board approved this prospective study, and all patients provided a written informed consent. From December 2016 to June 2018, 482 consecutive female patients with 490 breast lesions who were referred to our hospital underwent US and SMI. Of these patients, 262 were malignant breast lesions. The following inclusion criteria were applied: 1) female patients older than 18 years of age; 2) patients for whom US and SMI screening were performed; 3) lesion size <6 cm (no more than the maximum scope of the probe display); 4) the pathological type was invasive breast cancer. The exclusion criteria were as follows: 1) lesions larger than the probe because the US parameter would be shielded; 2) Patients who received treatment like biopsy, surgery, or neoadjuvant chemotherapy were excluded because these treatments may have altered the blood supply of the breast lesions; 3) Pregnant women were excluded because breast parenchymal changes can also alter the blood flow to the targeted lesions. All patients underwent excision biopsy and were histopathologically examined. A total of 225 patients were finally included; the study flowchart is shown in **Figure 1**. The final pathologic results were considered the diagnostic gold standard. The clinical features of the patients were recorded.

### Ultrasound and Superb Microvascular Imaging Examination

All lesions were detected using the US Aplio 500 (L14-5, Aplio 500, Canon Medical Systems Corporation, Tokyo, Japan) that could perform US and SMI examination. One radiologist (HW with >15 years of ultrasonic work experience and 2 months of experience in SMI) conducted US and SMI examinations. First, US images of the lesions were obtained, including B-mode and color Doppler images. The tumor size, shape, echogenicity, margin, presence of architectural distortion, acoustic shadowing, microcalcifications, and vascularity were evaluated by conventional US. After conventional US, SMI images were obtained by the same radiologists. SMI examination was performed using color mode. The parameters of the SMI were set to a low-velocity range (1.2~1.6 cm/s) to visualize extremely low-velocity flow with good resolution and a high frame rate with minimal flash artifacts (frame rate, 25~30/s; pulse repetition frequency, 15.4~20.2 kHz; dynamic range, 21 dB). The range of depth was adjusted to 2.5~6 cm according to lesion size, and the detectable width of the linear probe was 6 cm. Three-dimensional (3-D) SMI vasculature volume could be reconstructed from 2-D SMI images scanned using 2-D linear transducers. The 3-D SMI was used as a qualitative guidance to identify the 2-D SMI plane with the most abundant vasculature. The VI (%) was automatically calculated by manually tracing the boundary of the breast lesion on the 2-D SMI image with the most abundant vasculature by a radiologist three times and then averaged. The total inspection time was about 15–20 min.

### Molecular Classification of Groups

ER, PR, and HER-2 levels were evaluated using immunohistochemistry (IHC). The Allred scoring system was used to assess ER and PR with a score of more than 2 points being considered positive (20). HER-2 expression was defined as positive when membrane 3+ and a 2+ were analyzed using fluorescence *in situ* hybridization (FISH) to determine a positive or negative status. In addition, Ki67 expression of 14% or more was considered positive, and Ki-67 index was stratified into three groups: “low” (<14%),”intermediate” (14%–20%), and “high” (≥20%) (21, 22). Molecular subtypes identified by St. Gallen International Expert Consensus (2) were as follows:

1. Luminal A: ER-positive and/or PR-positive, HER-2-negative, Ki-67 low (<14%);
2. Luminal B HER-2-negative: ER-positive and/or PR-positive, HER-2-negative, and Ki-67 high (≥14%);
3. Luminal B HER-2-positive: ER-positive and/or PR-positive, HER-2-positive, and any Ki-67 index;
4. HER-2-positive: ER-negative, PR-negative, and HER-2-positive;
5. Triple-negative: ER-negative, PR-negative, and HER-2-negative.

### TNM Stage

TNM staging, published by the American Joint Committee on Cancer, uses both clinical and pathologic information of tumor size (T), status of regional lymph nodes (N), and distant metastases (M). The staging combines these factors and stratifies the disease into one of 5 stages (0, I, II, III, and IV) (23).

### Nuclear Grade

Modified Bloom Richardson grading system was used for grading the tumors as grades 1, 2, and 3 (24).

### Statistical Analysis

Kolmogorov–Smirnov test was used to test the normality of quantitative data. The quantitative data of normal distribution were expressed in means and standard deviations, and *t* test was used for the comparison between the two groups. The non-normality quantitative data were expressed in median (P25~P75), and the Mann–Whitney U rank sum test was used for the comparison between the two groups. The qualitative data were presented as frequencies. The correlations between the VI of the breast cancer and the clinical, pathological, and immunohistochemical data were evaluated using the Mann–Whitney U rank sum test (two variables), the Kruskal–Wallis test (three or more nominal variables), linear-by-linear association test (three or more ordered variables), and linear regression. Multiple regression analysis was used to determine the clinicopathological and immunohistochemical variables that were independently associated with VI (The VI values were transformed into logarithm). The significant difference in median VI among the five subgroups was calculated using single-factor analysis of variance and a multiple comparison test for parametric data with Bonferroni correction. Receiver operating characteristic (ROC) curve analysis was performed to examine which subgroups could be differentiated from the others on the basis of VI. The diagnostic performance of the optimal cutoff value for differentiating one subgroup from the others was also determined by ROC analysis. All statistical analyses were conducted using SPSS software version 20.0 (IBM, Armonk, NY, USA). Differences with *P* < 0.05 were considered statistically significant.

## Results

A total of 482 patients with 490 breast lesions were screened by US and SMI. The final analysis included 225 patients with invasive breast cancers (mean age: 51.3 ± 12.2 years, range 23~83 years). The mean size of the invasive tumors was 2.6 ± 1.4 cm (range 0.4~5.9 cm). The histological classifications of the cancers were as follows: invasive ductal carcinoma (IDC; 210 patients, 93.3%), invasive lobular carcinoma (4 patients, 1.8%), and other specified cancers (11 patients, 4.9%; six mucinous carcinomas, two invasive solid papillary carcinoma, one micro invasive solid papillary carcinoma, one invasive encapsulated papillary carcinoma plus IDC, one sarcoma). The molecular subtypes of the 225 tumors were luminal A in 41 patients (18.2%), luminal B HER-2-negative in 91 patients (40.4%), luminal B HER-2-positive in 26 patients (11.6%), HER-2-positive in 17 patients (7.6%), and triple-negative in 50 patients (22.2%). The clinicopathological findings and results of univariate regression analysis for the 225 breast cancers are summarized in **Table 1**.

**Table 1:** Correlations between clinicopathological and vascular index (VI) values of breast cancers.

	Number	VI (%)M (25%~75%)	*Z*	*P* value
Size(cm)			-0.819	0.413
≤2	94	7.7 (4.0~13.4)		
>2	131	7.0 (4.4~11.3)		
**ER status**			-2.361	**0.018**
Positive	158	6.7 (4.0~10.8)		
Negative	67	8.8 (5.1~18.2)		
PR status			-1.835	0.066
Positive	134	6.6 (3.9~10.8)		
Negative	91	8.2 (4.4~14.2)		
HER-2 status			-0.516	0.606
Negative	181	7.4 (4.3~12.1)		
Positive	44	6.7 (4.1~11.6)		
Ki-67 index (%)			-1.650	0.099
<14	45	5.9 (2.7~11.3)		
≥14	180	7.6 (4.4-12.4)		
Ki-67 index (%)			5.563	0.062
<14	45	5.9 (2.7~11.3)		
14~20	20	6.8 (2.6~9.3)		
≥20	160	7.7 (4.5~12.7)		
**Ki-67 index (%)**			-2.322	**0.020**
<20	65	6.2 (2.7~10.8)		
≥20	160	7.7 (4.5~12.7)		
Histology				
Invasive ductal carcinoma	210	7.5 (4.3~12.1)	2.931	0.231
Invasive lobular carcinoma Others	4 11	5.4 (1.5~12.0) 4.8 (1.4~9.5)		
**Nuclear grade**			6.792	**0.034**
1	32	5.3 (1.8~9.6)		
2	101	7.3 (4.3~12.4)		
3	92	8.2 (4.5~13.7)		
Axillary lymph node metastasis			-0.009	0.992
Absent	138	7.6 (3.8~13.3)		
Present	87	7.0 (4.7~10.6)		
Stage			2.646	0.449
I	66	8.5 (4.1~13.8)		
II	137	7.0 (4.3~11.4)		
III	21	6.5 (4.6~11.8)		
IV	1	11.7		

VI (%)M (25%~75%), Vascular index (%) Median (25%~75%); ER, estrogen receptor; HER-2, human epidermal growth factor receptor-2.

The difference is statistically significant in bold.

ER negativity (Z = -2.166, *P* = 0.031), higher nuclear grade (Z = 6.792, *P* = 0.034), and higher Ki-67 index (≥20%) (Z = -2.322, *P* = 0.020) were significantly associated with higher VI value, whereas the tumor size, PR status, HER-2 status, histology, axillary lymph node metastasis, and TNM stage were not associated with VI value significantly (**Table 1**). VI decreased with the increase of tumor size of the infiltrative breast cancer. The median VI values were 5.9% (2.6%~11.6%), 7.3% (4.4%~10.5%), 6.3% (3.9%~11.3%), 8.2% (4.9%~15.6%), and 9.2% (5.1%~15.3%) for the luminal A, luminal B HER-2-negative, luminal B HER-2-positive, HER-2-positive, and triple-negative subgroups, respectively (**Table 2**). The VI did not differ significantly among the five subgroups (*F* = 1.855, *P* = 0.119).

**Table 2:** VI in different molecular subgroups of breast cancer.

Subgroup	Number (%)	VI (%)	
		M (25%~75%)	Range
Luminal A	41 (18.2)	5.9 (2.6~11.6)	0~47.1
Luminal B HER-2-negative	91 (40.4)	7.3 (4.4~10.5)	0~29.5
Luminal B HER-2-positive	26 (11.6)	6.3 (3.9~11.3)	0.6~22.2
HER-2-positive	17 (7.6)	8.2 (4.9~15.6)	0.9~54.4
Triple-negative	50 (22.2)	9.2 (5.1~15.3)	0.7~32.9
Total	225 (100)	7.3 (4.2~11.8)	0~54.4

Single-factor analysis of variance and Bonferroni correction showed no significant differences among the five subgroups (F = 1.855; P = 0.119) and any two subgroups (P > 0.05). VI (%)M (25%~75%), Vascular index (%) Median (25%~75%); ER, estrogen receptor; HER-2, human epidermal growth factor receptor-2.

Multiple regression analysis was performed to select independent clinicopathological variables associated with VI in all patients with primary invasive breast cancer. The variables entered into the multivariate models included tumor size (≤2 cm vs. >2 cm), ER status, and Ki-67 expression. Backward regression analysis showed that tumor size, ER status, and Ki-67 index independently influenced VI (**Table 3**).

**Table 3:** Multiple regression analysis showing the effect of different characteristics on VI.

Factor	Favorable	Unfavorable	*P* value	β	t value	Lower 0.95	Upper 0.95
**ER**	Positive	Negative	**0.046**	-0.288	-2.007	-0.571	-0.005
**Ki-67**	<20%	≥20%	**0.035**	0.355	2.120	0.025	0.684
**Size**	>2cm	≤2cm	**0.023**	-0.306	-2.287	-0.570	-0.042

ER, estrogen receptor.

The median VI was 5.9% (2.6%~11.6%) (range 0%~47.1%) for luminal A tumors (n = 41, 18.2%) and 7.4% (4.2%~11.7%) (range 0~54.4%) for non-luminal A tumors (n = 184, 81.8%) (*P* = 0.1059). A cutoff VI of 4.1% yielded a sensitivity of 79.9% [95% confidence interval (95% CI), 73.4%~85.4%], a specificity of 41.5% (95% CI, 26.3%~57.9%), positive predictive value (PPV) of 86.0% (95% CI, 82.4%~88.9%), negative predictive value (NPV) of 31.5% (95% CI, 22.4%~42.2%), an accuracy of 72.9%, and an AUC of 0.58 (95% CI, 0.51~0.65) for differentiation of luminal A from non-luminal A subtypes (Z = 1.507, *P* = 0.0315). The positive likelihood ratio was 2.065 (95% CI, 0.9~1.6) (**Table 4**).

**Table 4:** Diagnostic performance of VI for luminal A, triple-negative, and HER-2-positive invasive breast cancers.

Molecular subtype	Cut point	Sensitivity (95% CI)	Specificity (95% CI)	PPV (95% CI)	NPV (95% CI)	ACC	AUC (95% CI)
**Luminal A**	4.1%	79.9% (73.4%~85.4%)	41.5% (26.3%~57.9%)	86.0% (82.4%~88.9%)	31.5% (22.4%~42.2%)	72.9%	0.58 (0.51~0.65)
**Triple-negative**	16.4%	30.0% (17.9%~44.1%)	90.3% (84.9%~94.24%)	46.9% (32.2%~62.1%)	81.9% (78.9%~84.5%)	76.9%	0.60 (0.53 ~ 0.67)
**HER-2**	5.3%	76.5% (50.1%~93.2%)	37.0% (30.4%~44.0%)	9.0% (7.0%~11.6%)	95.1% (88.9%~97.9%)	40.0%	0.55 (0.48~0.62)

HER-2, human epidermal growth factor receptor-2.

The median VI was 8.2% (4.9%–15.6%) (range 0.9%~54.4%) for HER-2-positive tumors (n = 17, 7.6%) and 7.3% (4.2%~11.7%) (range 0~47.1%) for non-HER-2-positive tumors (n = 208, 92.4%) (*P* = 0.5052). A cutoff VI of 5.3% yielded a sensitivity of 76.5% (95% CI, 50.1%–93.2%), a specificity of 37.0% (95% CI, 30.4%~44.0%), PPV of 9.0% (95% CI, 7.0%~11.6%), NPV of 95.1% (95% CI, 88.9%~97.9%), an accuracy of 40%, and an AUC of 0.55 (95% CI, 0.48~0.62) for prediction of HER-2-positive tumors (Z = 0.659, *P* = 0.5099). The positive likelihood ratio was 1.214 (95% CI, 0.9~1.6).

The median VI was 9.2% (5.1%~15.3%) (range 0.7%~32.9%) for triple-negative tumors (n = 50, 22.2%) and 6.8% (4.1%–10.9%) (range 0%~54.4%) for non-triple-negative tumors (n = 175, 77.8%) (*P* = 0.0298). A cutoff VI of 16.4% yielded a sensitivity of 30.0% (95% CI, 17.9%~44.1%), a specificity of 90.3% (95% CI, 84.9%~94.24%), PPV of 46.9% (95% CI, 32.2%~62.1%), NPV of 81.9% (78.9%~84.5%), an accuracy of 76.9%, and an AUC of 0.60 (95% CI, 0.533–0.665) for prediction of triple-negative tumors (Z = 2.151, *P* = 0.0315). The positive likelihood ratio was 3.093 (95% CI, 1.7~5.7).

## Discussion

The major findings of the present study were as follows: 1) VI shows a certain degree of correlation with the molecular subtype in invasive breast cancer; 2) ER negativity, higher tumor grade, and higher Ki-67 index (≥20%) were significantly associated with a higher VI value; (3) Tumor size, ER status, and Ki-67 index were shown to independently influence VI; (4) VI was of value in predicting the luminal A with high sensitivity and PPV and triple-negative type with high specificity and NPV.

Recent studies confirmed the predictive value of microvascular imaging features in the differentiation of breast tumors; malignant breast tumors have a higher VI than benign tumors, and VI could help distinguish malignant from benign breast tumors (16, 17, 19, 25). Accurately assessing the blood flow status in tumor can provide a basis for judging the malignancy of tumors. Tumor angiogenesis is variable according to the hormone receptor status and molecular subtype of breast cancer (26). The 3-D power Doppler sonographic vascular features are associated with the molecular subtypes and tumor grades in breast cancer; differences in 3-D power Doppler vascular features among subtypes of IDCs are attributed to the ER status (27). Malignant masses negative for ER or positive for Ki67 had higher microvessel density (MVD) (17). VI was significantly correlated with MVD (17, 28). Our results showed that ER negativity, higher nuclear grade, and higher Ki-67 index (≥20%) were significantly associated with higher VI value in invasive breast tumors, as reported in literature (17). It may be because ER inhibits tumor angiogenesis pathway resulting in decreased tumor vascular proliferation and perfusion. Ki-67 is a nuclear protein being associated with cellular proliferation. Ki-67 plays an important role in the process of cell proliferation and has a positive correlation with vascular endothelial growth factor (VEGF), which could promote angiogenesis. This also causes a mass to grow faster and increase in size with a higher degree. In the condition of high expression of Ki-67, the proliferating cells are accompanied by new blood vessels, with the blood vessel density increasing, resulting in a rich blood flow, a high color Doppler flow imaging (CDFI) grade, and a higher VI (29). A mass with diameter >2 cm is positively correlated with high Ki-67 (30), thus the more tumor vasculature. However, VI is defined as the ratio of Doppler signal pixels to pixels in the total lesion, so in the present study, VI decreased with the increase of tumor size of the infiltrative breast cancer.

**Figure 2:**
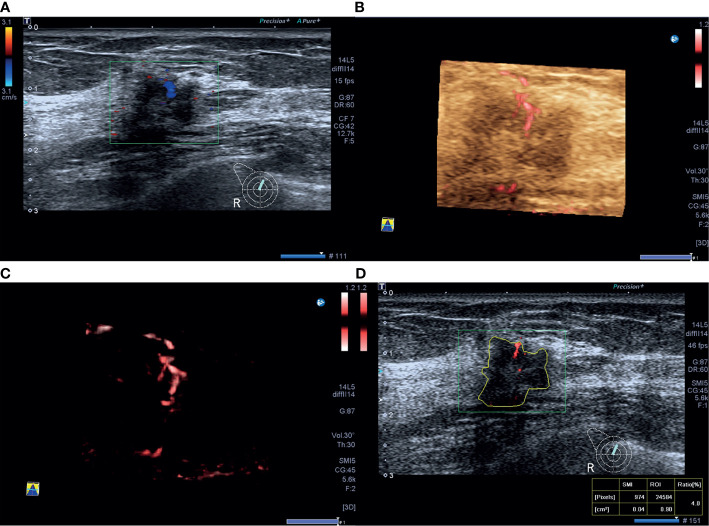
A 46-year-old woman with luminal A invasive ductal cancer [1.7 cm, ER 90%, PR 95%, HER-2(-), Ki-67 10%, nuclear grade 1, T1N0M0]. **(A)** Color Doppler flow imaging image shows linear blood flow signals. **(B, C)** Smart three-dimensional superb microvascular imaging reveals linear blood flow. **(D)** Vascular index was measured on the plane containing the most abundant vasculature with a value of 4.0%.

**Figure 3:**
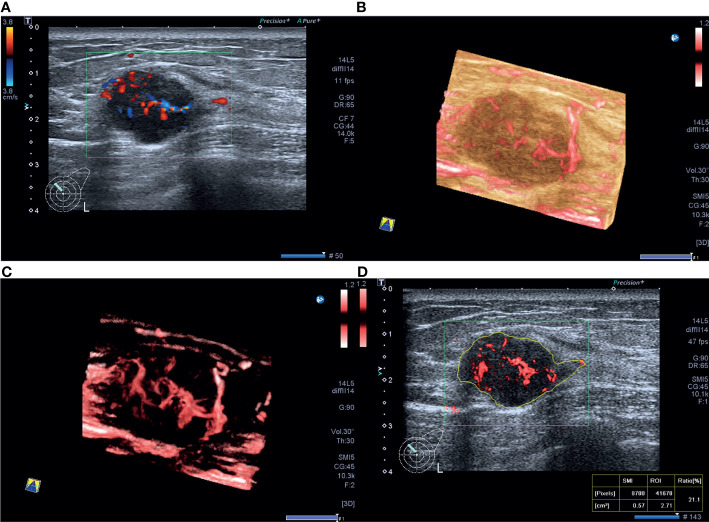
A 40-year-old woman with triple-negative invasive ductal cancer [2.3 cm, ER (-), PR (-), HER-2(-), Ki-67 95%, nuclear grade 2, T2N1M0]. **(A)** Color Doppler flow image shows abundant and disordered blood flow signals. **(B, C)** Smart three-dimensional superb microvascular imaging reveals detailed and abundant vascular architecture with crab claw-like blood flow. **(D)** Vascular index was measured on the plane containing the most abundant vasculature with a value of 21.1%.

VI was not significantly correlated with all the molecular subtypes of invasive breast cancer, this may be due to that one of the most important limitations in this study was that ER, PR, and HER-2 levels were evaluated using IHC. We know very well that this determination represents a surrogate and cannot establish the intrinsic subtype of any given cancer while the correct correlation should have been assessed by genomics (31), with discordance rates between IHC‐based markers and gene‐based assays as high as 30% (32). Another reason may be due to the high heterogeneity of vasculature in invasive breast cancer, and there is considerable vasculature overlap among different molecular subtypes in invasive breast cancer. The luminal A tumors had lower VI values compared to non-luminal A tumors in the present study, consistent with the study that reported that the luminal A subtype was composed of masses with low vascularity (33) (**Figure 2**). Furthermore, vascular features including the number of vascular trees, total vessel length, number of bifurcations, and vessel-to-volume ratio in luminal type were significantly lower compared to HER-2-enriched or triple-negative types (27). HER-2-enriched cancers more commonly present as Adler grades 2 and 3 on ultrasonography (73.3%) (34). Here, 30.0% TNBCs had abundant blood supply on SMI images in the present study (**Figure 3**), consistent with the previous reports that 32.9%–43.4% TNBCs showed hypervascularity or Adler grades 2 and 3 on color Doppler flow imaging (34, 35). VI had good performance in predicting luminal A type with high sensitivity and PPV and triple-negative type with high specificity and NPV in the present study.

The present study has a few limitations. First, this study included a limited number of patients. Thus, VI value did not reflect the vascularity of all the invasive breast tumors. Second, VI value did not reflect the overall vascularity of the breast lesion. Since the measurements of VI were obtained on a 2-D SMI plane with the abundant vasculature although under the guidance of 3-D SMI, it was impossible to quantify the total volumetric vascularity of the lesion.

## Conclusions

In conclusion, VI, as a quantitative index obtained by SMI examination, could reflect histologic vascular changes in invasive breast cancer and was found to be higher in more biologically aggressive breast tumors. VI shows a certain degree of correlation with the molecular subtype in invasive breast cancer and plays a limited role in predicting the luminal A and triple-negative subtype.

## Data Availability Statement

The original contributions presented in the study are included in the article/supplementary material. Further inquiries can be directed to the corresponding authors.

## Ethics Statement

The Ethics Committee of Peking Union Medical College Hospital approved this prospective study. All patients were aware of the examination process and provided written informed consent. There is no identifiable patient information. The patients/participants provided their written informed consent to participate in this study.

## Author Contributions

Conceptualization, HW; Data curation, XZ, SC, and LZ; Formal analysis, XZ and HW; Investigation, HW; Resources, HW, QZ, JL, QS, and YJ; Writing original draft, XZ. All authors contributed to the article and approved the submitted version.

## Funding

This work is supported by the Beijing Natural Science Foundation (7202156) and the Teaching Reform Project of Peking Union Medical College (10023201900113).

## Conflict of Interest

The authors declare that the research was conducted in the absence of any commercial or financial relationships that could be constructed as a potential conflict of interest.

## Publisher’s Note

All claims expressed in this article are solely those of the authors and do not necessarily represent those of their affiliated organizations, or those of the publisher, the editors and the reviewers. Any product that may be evaluated in this article, or claim that may be made by its manufacturer, is not guaranteed or endorsed by the publisher.

## Abbreviations

TNBCs, triple-negative breast cancers; ER, estrogen receptor; PR, progesterone receptor; HER-2, human epidermal growth factor receptor-2; FISH, fluorescence *in situ* hybridization; IDC, invasive ductal carcinoma; MVD, microvessel density; PPV, positive predictive value; NPV, negative predictive value; ACC, accuracy; AUC, area under the curve.
